# A pilot study of essential tremor: cerebellar GABA+/Glx ratio is correlated with tremor severity

**DOI:** 10.1186/s40673-020-00116-y

**Published:** 2020-06-26

**Authors:** Sofie Tapper, Nathanael Göransson, Peter Lundberg, Anders Tisell, Peter Zsigmond

**Affiliations:** 1 Center for Medical Image Science and Visualization (CMIV), Linköping University, Linköping, Sweden; 2 Department of Medical Radiation Physics and Department of Health, Medicine and Caring Sciences, Linköping University, Linköping, Sweden; 3 Department of Biomedical Engineering, Linköping University, Linköping, Sweden; 4 Department of Neurosurgery and Department of Biomedical and Clinical Sciences, Linköping University, Linköping, Sweden

**Keywords:** Essential tremor, GABA, Glutamate, Magnetic resonance spectroscopy, MEGA-PRESS

## Abstract

**Objective:**

Essential tremor is a common movement disorder with an unclear origin. Emerging evidence suggests the role of the cerebellum and the thalamus in tremor pathophysiology. We examined the two main neurotransmitters acting inhibitory (GABA+) and excitatory (Glx) respectively, in the thalamus and cerebellum, in patients diagnosed with severe essential tremor. Furthermore, we also investigated the relationship between determined neurotransmitter concentrations and tremor severity in the essential tremor patients.

**Methods:**

Ten essential tremor patients (prior to deep brain stimulation surgery) and six healthy controls, were scanned using a 3 T MR system. GABA+ and Glx concentrations were measured using magnetic resonance spectroscopy (MRS) performed using single voxel MEGA-PRESS. For the purpose of assessing the tremor severity, the essential tremor rating scale (ETRS) was used in accordance with Fahn, Tolosa, and Marin.

**Results:**

We demonstrated that the cerebellar GABA+/Glx ratio was positively correlated to the ETRS (r = 0.70, p = 0.03) in essential tremor. Cerebellar and thalamic GABA+ and Glx concentrations did not show any significant difference when comparing essential tremor patients with healthy controls, at the group level.

**Conclusion:**

We demonstrated a positive correlation between increasing tremor disability and the ratio of GABA+/ Glx in the cerebellum of essential tremor patients. This highlights the impact of an altered balance of the excitatory and inhibitory neurotransmitters in tremor severity. Rather than a change in GABA+, which was constant, we attribute this finding to an overall decrease of Glx.

## Introduction

Essential tremor (ET) is one of the most common movement disorders with a prevalence of 4.6% in patients over 65 years old [1]. The diagnosis of ET is generally based on a thorough analysis of clinical history, including a neurological examination by a movement disorder specialist. The upper limbs are affected in 95% of patients, that result in shaking of the hands and forearms during simple voluntary movements [2, 3]. In addition, a wide range of other symptoms can appear (cognitive, psychiatric and other motor symptoms); the clinical diversity makes ET a very heterogenic disease [3, 4]. Not surprisingly, the pathophysiology of ET is therefore still not completely understood [5].

Recent advances in neuroimaging and spectroscopy opens up for the study of a novel range of structural, functional and metabolic changes in these patients [5]. Resting state functional MRI (fMRI) studies have shown decreased functional connectivity of the cerebellar network in ET [6]. Also significant alterations have been observed in the cerebello-thalamo-cortical network in patients with ET, which correlated with the overall tremor severity [7]. In addition, this network is the most common target for deep brain stimulation (DBS) surgery in ET. Several imaging studies support a cerebellar involvement in ET, where for example alterations in the grey matter volume of several brain regions including the cerebellum in ET has been observed [8]. An fMRI study showed an association between tremor and ipsilateral cerebellar activity [9], and a ‘positron emission tomography’ (PET) study showed an increased cerebellar blood flow in ET [10]. These and other studies suggests that both the cerebellum and the thalamus play a central role in the pathophysiology of ET [11–13].

Postmortem studies have shown structural changes in the cerebellum of patients with ET [14–16], including a reduced number of ‘Purkinje cells’ (PCs), dendritic swelling and increased Purkinje cell torpedoes in the cerebellum [14] (i.e., neurodegeneration). PCs propagate an input to the deep cerebellar nuclei, dentate nucleus, through gamma-aminobutyric acid (GABA) which is an inhibitory neurotransmitter. A postmortem study showed a decreased number of GABA receptors in the dentate nucleus, GABA_A_ (35% reduction) and GABA_B_ (ca. 25% reduction) [17]. Moreover, PET studies using Flumazenil, which specifically binds to the GABA_A_ receptor have shown an increased binding in the cerebellum, but also in the thalamus and the premotor cortex [18]. Moreover, the severity of tremor has been correlated to abnormalities found in the GABA receptor binding [19], and several GABA-ergic drugs have been shown to relieve ET [20].

Climbing fibers (CF) of the cerebellum use the excitatory neurotransmitter glutamate (Glu) (see Fig. 4), and anomalies of CF-PC synaptic connections with decreased climbing fiber synaptic density in the cerebellar cortex have also been observed [15]. An altered distribution of climbing fibers and synapses extending into the outer 20% of the molecular layer, ‘Parallel fiber’ (PF) territory, has been reported [15, 16], and it has been shown that tremor severity correlates inversely with synapse CF-PC abnormalities in the PF area [21].

‘Proton magnetic resonance spectroscopy’ (^1^H-MRS) is a tool for non-invasive metabolite quantification, including *N*-acetyl-L-aspartate (NAA), total choline (tCho), and total creatine (tCr). It is therefore a powerful tool to quantitatively investigate the biochemistry and metabolism in the human brain in relation to both pathologies and healthy conditions. Greater magnetic field strength (such as 3 T instead of 1.5 T) allows for an increased signal-to-noise-ratio, better temporal resolution, and also provides a larger spectral dispersion which significantly improves quantification [22]. The latter allows for the detection of metabolites of lower concentrations than otherwise.

Louis et al. investigated the GABA concentrations in large voxels including the dentate nucleus by the use of MRS. Interestingly they found that the *right* dentate nucleus GABA concentration was significantly higher than the GABA concentration obtained from the *left* side, only observed for the ET patients [23].

There seems to be a consensus about the involvement of the cerebellum and thalamus in ET, although the potential neurochemical abnormalities causing ET remain to be further elucidated. Our aim in this study was therefore to evaluate the relationship between glutamate (Glu, determined as Glx) and GABA (determined as GABA+) concentrations in the cerebellum and thalamus, in patients diagnosed with severe ET immediately prior to DBS intervention, and to compare the concentrations of these with those obtained by examining a healthy control group. Furthermore, we aimed to investigate the correlation between neurotransmitter concentrations in the ET group and the tremor severity as determined using an ET rating scale (ETRS) [24], in order to facilitate further conclusions with respect to the underlying pathology, relationship to debilitating tremor severity and metabolic alterations.

## Materials and methods

This study was approved by the local ethics committee at the University Hospital in Linköping (Ref. No. 2013/403–31, P. Zsigmond), and informed written consent was received from the patients.

### Subject description

Ten right-handed Caucasian ET patients (60.2 ± 9.7 years, seven males, Table 1), and six right-handed Caucasian healthy controls (HCs) (62.2 ± 11.4 years, five males) were included in this study. The patients were referred to the neurosurgical department in Linköping after being assessed and diagnosed by a movement disorder specialist. All anti tremor medication was withdrawn one week prior to MR-examination.

**Table 1:** Description of the ET patients. Each patent’s age and sex, history of ET, if treated with medication, if a current smoker, and the weekly ethanol intake are summarized. The ETRS score, thus the sum of the evaluated items, is also shown for each patient

Patient	Age	Sex	History of ET	Medication	Smoker	Ethanol	ETRS score
1	51	M	> 20 years	0	No	< 1 drink/week	21
2	76	M	> 10 years	0	No	< 1 drink/week	14
3	67	M	> 10 years	0	No	< 1 drink/week	23
4	67	M	> 10 years	0	No	< 1 drink/week	16
5	41	M	10 years	0	No	< 1 drink/week	15
6	59	F	8 years	0	Yes	1 drink/day	21
7	69	F	4 years	0	No	< 1 drink/week	17
8	60	M	> 15 years	0	No	< 1 drink/week	17
9	63	F	> 20 years	0	No	< 1 drink/week	15
10	53	M	> 20 years	0	No	< 1 drink/week	17

To assess the tremor severity in the patients and controls, the ‘essential tremor rating scale’ (ETRS) was used in accordance with Fahn, Tolosa, and Marin [24]. Only the dominant extremity was evaluated since this is the clinical routine in the department. Thus, the right upper extremity was evaluated with part A item 5 and part B items 10–14. The ETRS score was defined as the total sum score of these evaluated items, and the maximal score was 32 points (Supplementary Table 1).

### Magnetic resonance measurements

**Fig. 1:**
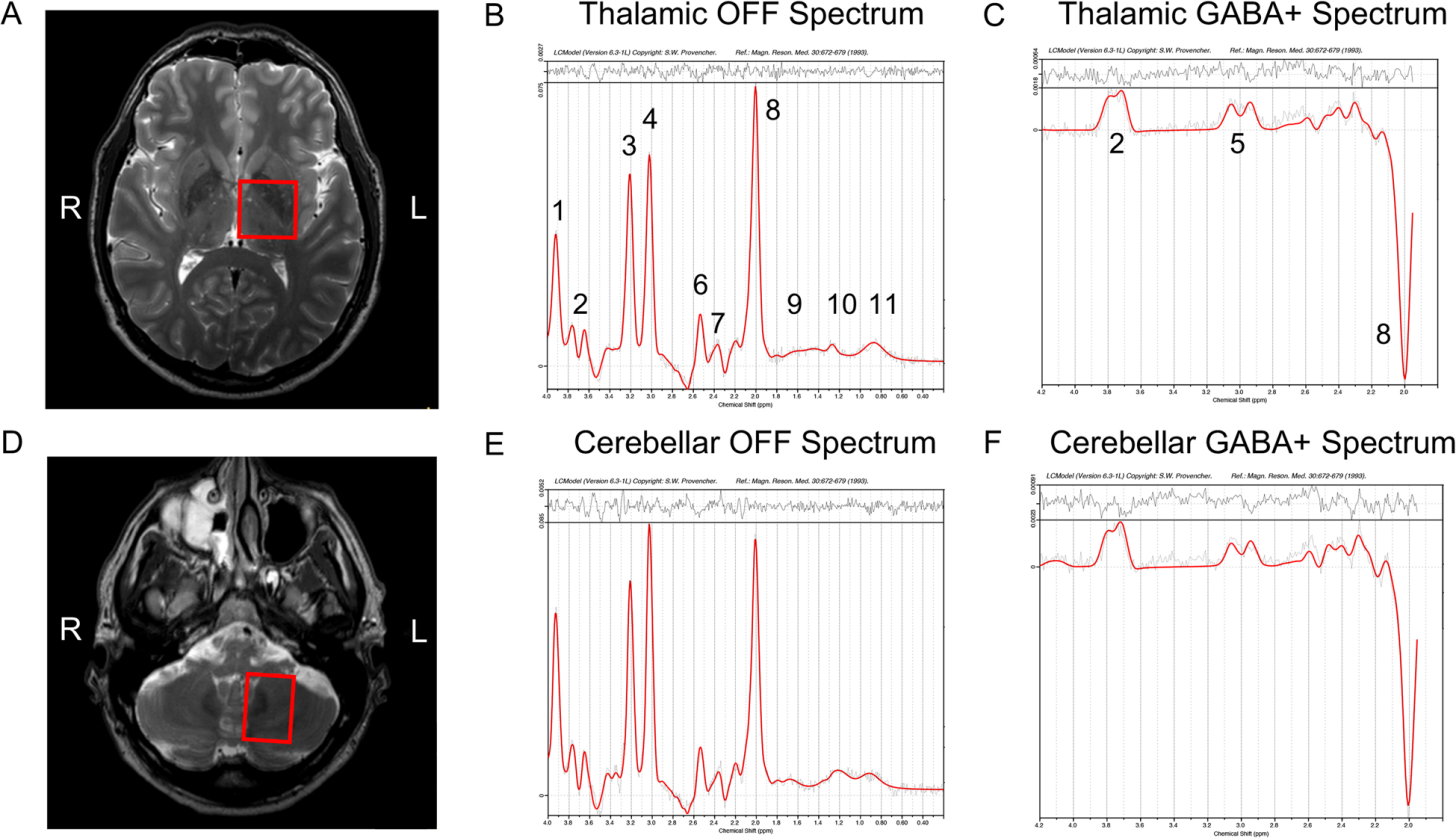
Representative voxel placements. Representative voxel placements (**a** and **d**) and corresponding thalamic (**b**-**c**) and cerebellar (**e**-**f**) spectra. Assignments: 1, Creatine (−^2^CH_2_-); 2, Glx (−^2^CH-); 3, Choline (−N (CH_3_)_3_); 4, Creatine (−N (CH_3_)); 5, GABA+ (−^4^CH_2_-); 6, tNA (−^3^CH_2_-); 7, Glx (−^4^CH_2_-); 8, tNA (−^2^CH_3_); 9–11, Macromolecules and lipids, −CH_2_-. Abbreviations: Glx, glutamate+glutamine; GABA+, γ-Aminobutyric acid (+macromolecule signal); tNA, total N-acetylaspartate (NAA + NAAG)

The ET patients and HCs were scanned using a 3 T Philips Ingenia MR system (Philips Healthcare, Best, Netherlands), equipped with a 12-channel phased array head coil. To assure correct voxel placements in the MRS measurements, T_2_-weighted turbo spin echo images were acquired in coronal (Res = 0.5*0.5*4 mm^3^, FOV = 230*230*96), sagittal (Res = 0.5*0.5*4 mm^3^, FOV = 242*242*96 mm^3^), and axial (Res: 0.5*0.5*2 mm^3^ FOV = 250*250*140 mm^3^) orientations. Furthermore, the MRS measurements were carried out using a single voxel MEGA-PRESS pulse sequence [25–27] optimized for GABA quantification (TR/TE = 2000/68 ms, editing pulse ON at 1.90 ppm, editing pulse OFF at 7.46 ppm, water suppression MOIST, eight phase cycle steps, 20 OFF and 20 ON dynamics, total acquisition time of 10 min and 40 s). A total of four MEGA-PRESS measurements were performed, two with the voxel (30 × 30 × 30 mm^3^, Fig. 1a) placed bilaterally in the thalamic region, and two with the voxel (35 × 25 × 25 mm^3^, Fig. 1d) placed bilaterally in the cerebellar region. The placement of the voxel in the anatomy was performed by two trained neurosurgeons ensuring a comparable placement among the ET cases and healthy controls. The reproducibility of the MEGA-PRESS measurements in cerebellar region was investigated and reported elsewhere [28]. After each MEGA-PRESS measurement, a shorter identical measurement but with unsuppressed water, was acquired to obtain a reference of water within the tissue in the voxel. This measurement was used for phase correction and water scaling of the resulting concentrations. Finally, since no macromolecular suppression was performed when acquiring the data in this study, the term GABA+ (GABA plus co-edited macromolecular signals) will be used throughout this report.

The MRS datasets were phase-corrected according to Klose [29] and frequency-aligned prior to averaging. For the purpose of GABA+ quantification, a resulting difference spectrum was computed by subtracting the averaged OFF spectrum from the averaged ON spectrum. Furthermore, due to the difficulty in separating glutamate from glutamine in the spectra, these concentrations are often reported as a combined measurement termed ‘Glx’. Only OFF data, which were unaffected by the GABA-editing pulses were used in the Glx quantification. After post-processing of the data, the difference spectra and averaged OFF spectra were quantified using LCModel (Version 6.3-1 L) [30], using simulated basis sets obtained from the Dydak Lab (December 14th 2018) [31, 32]. Figure 1 shows representative thalamic (B-C) and cerebellar (E-F) spectra fitted by LCModel. Finally, as a quality assurance, all spectra were visually inspected after LCModel fitting.

### Statistical analyses

Since the investigated subjects were not considered to be normally distributed, a non-parametric approach was used in the statistical analyses. As previously described, the main focus of this report was on the cerebellar and thalamic GABA+ and Glx concentrations acquired from the left and right hemispheres. However, to increase the power of the measurements, a bilateral measurement, thus, an average of the left and right computed concentrations was also computed and investigated. Furthermore, the GABA+/Glx ratio was also investigated to exclude any potential issues that might result from the water scaling.

First, the unpaired non-parametric Mann-Whitney test was used to compare the computed concentrations and ratios obtained from the ET patient group to the resulting concentrations obtained from the healthy controls. Second, due to a previous reported cerebellar GABA+ asymmetry [23], the Mann-Whitney test was also used to investigate a potential thalamic or cerebellar asymmetry of GABA+ in the ET patient group. Third, correlation analyses were performed, correlating the GABA+ and Glx concentrations, and the GABA+/Glx ratio to the computed ETRS score defined previously. These correlation analyses were only performed for the ET patient group, and the Spearman correlation coefficients and corresponding p-values were computed to show significance. Finally, a significance level of p = 0.05 was chosen for all analyses. No correction for multiple comparsions was applied.

## Results

The ET patients and HCs were similar in demographic factors and in clinical variables such as ethanol intake and handedness. Table 1 shows the ETRS score for each patient, and Supplementary Table 1 summarizes the score from each ETRS item for each patient, evaluated for the patients’ right upper extremity. The mean computed ETRS score over the ET patient group was 17.6 ± 2.9, which indicates a high overall tremor severity of this patient population. Meanwhile, all subjects in the healthy control group had an ETRS score of 0.

### GABA+ and Glx concentrations

One of the patient’s Glx-measurements (from the right thalamic voxel) was discarded after quality inspection of the fitted spectra. Therefore, the corresponding bilateral measurement was computed using only the left thalamic Glx concentration. All other spectra passed this visual inspection, and corresponding computed concentration were used in further analyses.

**Fig. 2:**
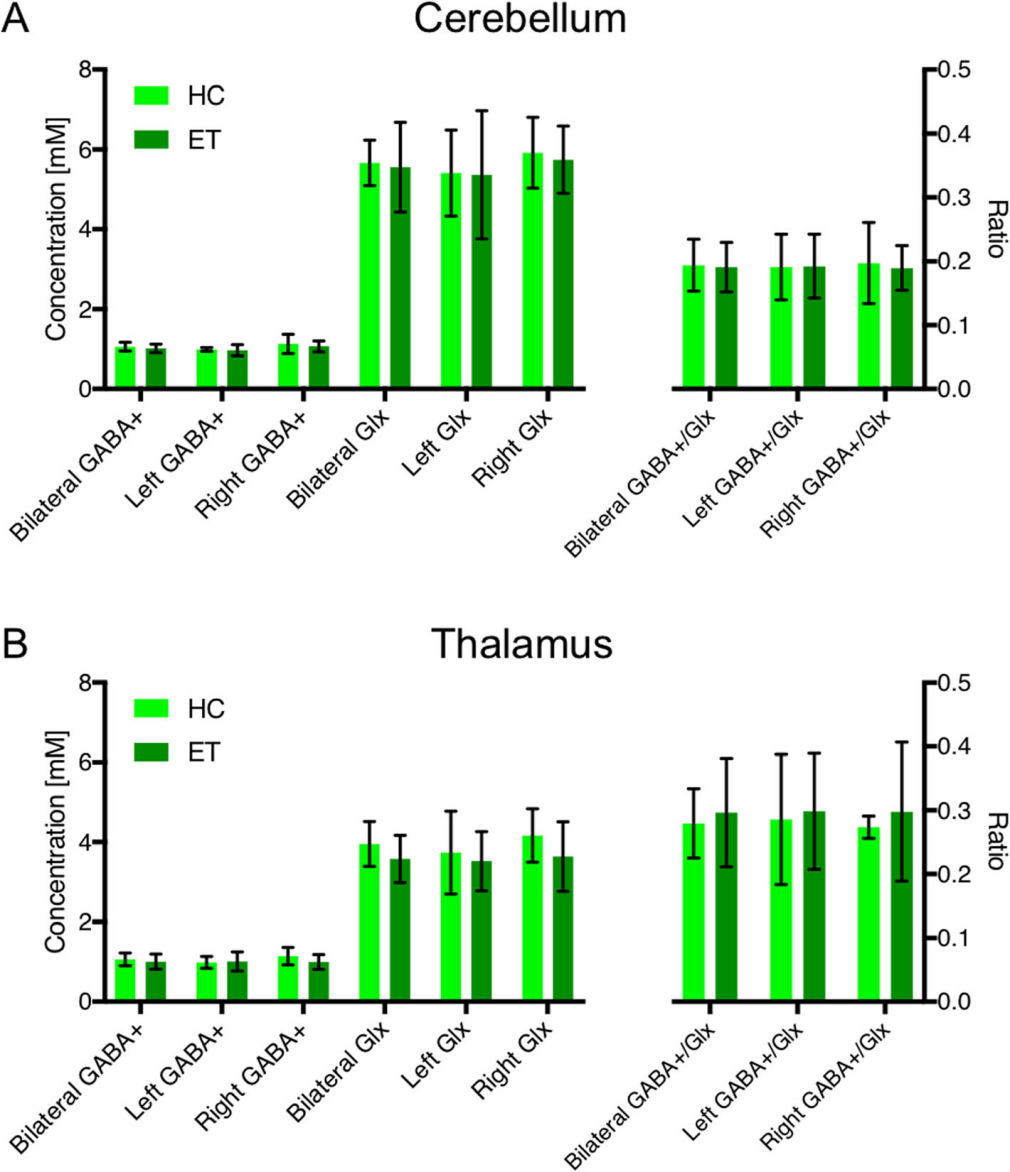
MRS Concentrations. Cerebellar (**a**) and thalamic (**b**) GABA+ and Glx concentrations, and GABA+/Glx ratios reported as a mean ± SD over the measurements. The healthy control group (HC) is shown in light green, and the ET patient group (ET) is shown in dark green

Figure 2 illustrates the average computed GABA+ and Glx concentrations, and also the GABA+/Glx ratios for the cerebellar (A) and thalamic (B) measurements. No significant differences between the ET and HC groups were observed in the bilateral, left and right GABA+ and Glx concentrations or in the corresponding GABA+/Glx ratios. Furthermore, a non-significant higher mean Glx concentration was observed in both the cerebellar and thalamic voxel placements for the HC group compared to the ET patients.

### Potential GABA+ asymmetry

We did not observe any significant differences between GABA+ concentrations in the left and right hemispheres (either for the thalamic (p = 0.85) or the cerebellar (p = 0.35) voxels, Supplementary Figure 1). However, generally a higher right cerebellar GABA+ concentration was observed, which agrees with previously reported results [23]. We did not observe any lateral differences of thalamic GABA+ concentrations.

### Bilateral MRS concentrations correlated to ETRS score

**Fig. 3:**
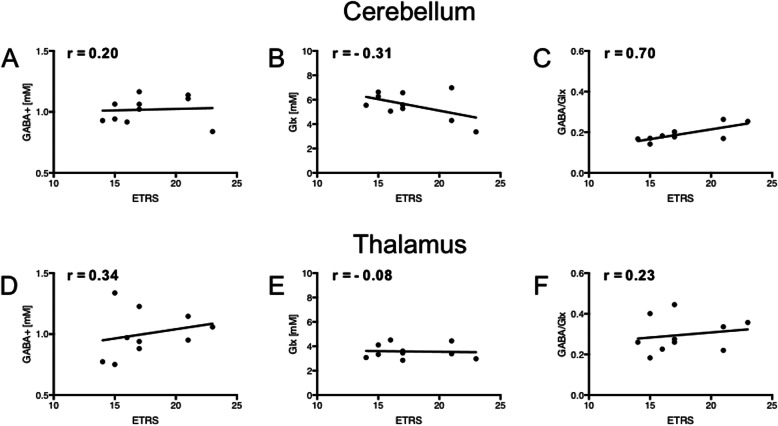
Concentrations related to ETRS. Correlation between concentration, or ratio, and ETRS score for the cerebellar (**a**-**c**) and thalamic (**d**-**f**) measurements. The Spearman correlation coefficient (r) is reported in each plot. The ratio between GABA+ and Glx was positively correlated with ETRS in the cerebellum (**c**), but not down-stream in the thalamus (**f**)

Bilateral cerebellar or thalamic GABA+ and Glx concentrations did not show any significant correlation to ETRS (Fig. 3, A-B and D-E). However, the cerebellar GABA+/Glx ratio was positively correlated to the ETRS (r = 0.70, p = 0.03, Fig. 3c). This positive correlation was due to the reduction in cerebellar Glx and a small increase in cerebellar GABA+ with a higher tremor severity. No significant correlation was observed between thalamic GABA+/Glx ratios and tremor severity (Fig. 3f).

### Cerebellar GABA+/Glx correlated to ETRS score

Since the bilateral GABA+/Glx ratio was positively correlated to the ETRS, the left and right sides are reported separately in Supplementary Figure 2. The right cerebellar GABA+/Glx ratio was significantly correlated to ETRS (r = 0.73, p = 0.02), whereas only a positive trend (r = 0.51, p = 0.14) was observed for the left side.

## Discussion

In the present study we have demonstrated the presence of neurometabolic changes in the cerebellum of patients with ET, which were correlated to ETRS. To our knowledge this is the first study to show a positive correlation between the ratio of GABA+ and Glx in the cerebellum of ET patients, and increasing tremor disability suggesting a pathological correlation. Our interpretation is that this can be attributed to an overall decrease of Glu, rather than an increase in GABA+, which was constant. This suggests that an increasing ETRS score is partly due to a disturbance in cerebellar Glu-concentration, and that there is a greater impact of low Glx concentration on tremor severity than a change of GABA+ concentration. Moreover, a greater ratio between the two main neurotransmitters acting in an inhibitory manner (GABA+) and an excitatory manner (Glx) on the synaptic level respectively, seems to correlate with a more severe tremor. This highlights the importance of the balance between the two excitatory and inhibitory neurotransmitters for proper motor control.

### Spatial origin of disease

**Fig. 4:**
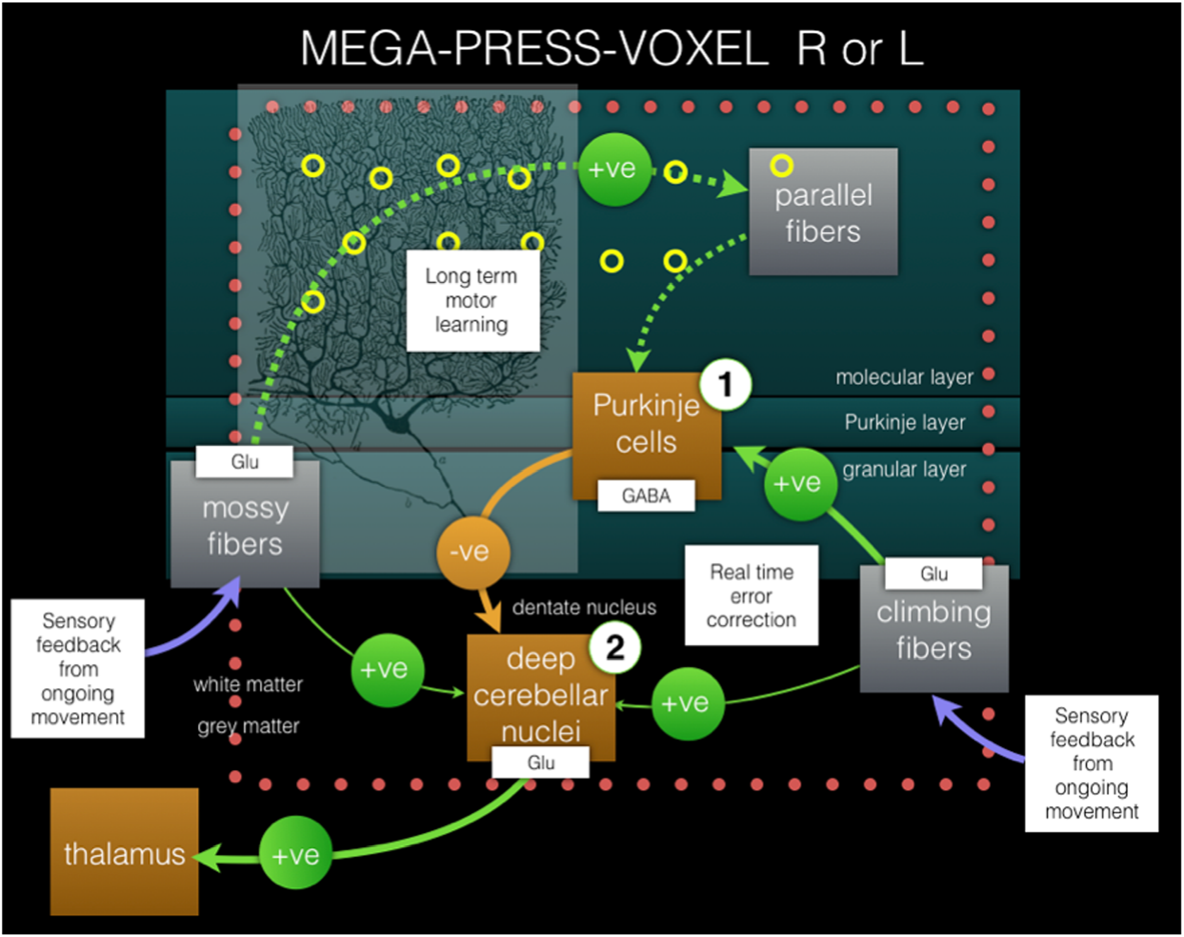
Pathways in ET. Pathways of cerebellar error-correction in ET. The suggested neurodegeneration results in a relative decrease of excitatory neurotransmitter Glu, resulting in a hypothetical net decreased inhibitory effect from Purkinje cells on the dentate nucleus in ET. This will in turn lead to an imbalance in the cerebellar error correction, based on a relative decrease of the effect induced by the excitatory feedback response from the sensory system of ongoing movement. The MEGA-PRESS voxels were acquired at 3 T, separately for the R and L cerebellum, including both the Purkinje layer, and the subcortical dentatus nucleus. The MEGA-PRESS-voxel (either R or L) is represented by a dotted square. Labels (1) and (2) represent two specifically reported locations of structural-functional aberrations in ET versus healthy controls (see Discussion for more details). Excitatory and inhibitory synaptic stimulation is represented by green and yellow circles, respectively. (The image of a Purkinje cell was obtained from Gray’s anatomy, 1918 edition, in the public domain)

Recent mechanistic research on ET has focused on anomalies localized in the cerebellum, in particular Purkinje cells and the dentate nucleus [33]; these sites are schematically labeled (1) and (2) in Fig. 4. Treatment of therapy resistant ET can include ‘Deep Brain Stimulation’ (DBS) of the ventral intermediate nucleus of the thalamus or the dentatorubrothalamic tract, and therefore both the cerebellum and thalamus were examined in this study. Moreover, the pathology in ET is not static, and the symptoms often progress and spread to other cerebellar areas [3]. In addition, previous reports have shown a correlation to increasing tremor severity with abnormalities; the latter included decreased cerebellar cortical NAA/tCr [34], CF-PC synaptic pathology [21], both suggesting cerebellar neurodegeneration. Also, increased thalamic Glx concentration and Glx/Cr ratio values [35] have both been shown to correlate to tremor severity. Our findings suggest an additional, previously unreported, perspective on the pathological process in the cerebellum.

We did not find a significant difference in GABA concentration (see Fig. 2), which is in accordance with previous studies, in which a reduction of the number of GABA receptors was observed, but not a reduction in the cerebellar GABA concentration [17]. MEGA-PRESS MRS at 3 T in our application did not allow us to spatially separate signals from the dentate nucleus and cortical Purkinje cells, which is illustrated schematically by the red dotted square in Fig. 4. Thus, we have to interpret the results as a combination of the outcome of cellular events within all of these tissues (see Fig. 1).

### Aspects of tissue structure

Tremor is the most prevalent movement disorder, but it represents a wide range of etiologies and it may represent a number of progressive diseases [36]. Moreover, clinical-, pathological and pharmacological heterogeneity may explain why evidence regarding etiology is difficult to conclude. A number of morphological aberrations have been identified in ET, mainly affecting Purkinje cells. In contrast, no pathological morphological changes have been found for other regions involved in the motor network (including the thalamus).

Previous observations include a reduced number of Purkinje cells (PCs), dendritic swelling and increased Purkinje cell torpedoes in the cerebellum [14], and an increased number of *heterotopic* Purkinje cells, localized in the molecular layer of the cerebellar cortex [37]. In contrast, a number of other studies have failed to show evidence of Purkinje cell loss [38, 39]. A difference in response to pharmacological substances further elucidates the heterogeneity of the disease.

### Is the disease symmetric, or not?

Similar to our observations, Louis and co-workers [23] did not find a reduction in GABA+ concentration in the dentate nucleus of ET using MEGA-PRESS, compared to the control subjects. Asymmetries of metabolic changes in ET have previously been reported, and Louis and co-workers did find an asymmetry, as the concentration of GABA in the right dentate nucleus was c. 15% larger than the concentration on the left dentate nucleus. This also agrees with our observations as we did not detect a GABA reduction, but an asymmetric distribution of GABA in the cerebellar region of interest. The latter was, in the patient cohort described here, limited to a trend (representing c. + 10% concentration difference of GABA+, R vs. L), and therefore was not a significant effect.

In the context of symmetry, it is important to consider aspects such as objective asymmetry in clinical expression, asymmetry in perceived disability (cf. dominant hand), as well as tissue asymmetry in terms of tissue structure and biochemical parameters. Contralateral low thalamic NAA/Cr ratio was found in ET patients with predominantly tremor of the right arm [40], and clinical asymmetry of tremor was correlated to a significant degree with cerebellar pathology [41]. All of our patients *perceived* more symptomatic tremor on the right side, thus displaying an asymmetry from a clinical perspective. This may explain why significant correlation of GABA+/Glx versus ETRS was only detected on the ipsilateral cerebellar side (see Supplementary Figure 2), although one potential limitation of this observation of the side-to-side difference may be the limited sample size.

### Inhibitory input

The Purkinje cells convey their input to the deep cerebellar nuclei through GABA, the main inhibitory neurotransmitter, which binds to synaptic GABA receptors. Flumazenil, which specifically binds to the GABA_A_ receptor, has been shown in PET studies to map the binding in the cerebellum (dentate nucleus), but also in the thalamus and the premotor cortex [18]. Other PET studies have also shown that the severity of tremor was correlated to abnormal GABA receptor binding [19]. In addition, a previous study showed a reduction of GABA receptors but not in GABA concentration in the cerebellum [17]. Available medication is very non-specific, but certain vectors that increase GABAergic transmission can be effective in treating ET, although with very variable outcomes [42].

Purkinje cells receive two direct excitatory inputs (Glu), from climbing fibers and from parallel fibers [43]. (See also Fig. 4) Notably, the synaptic distributions of the climbing fibers appear to be important to control Purkinje cell physiology and therefore normal cerebellar function. A particularly interesting finding, which affects the climbing fiber to Purkinje cell synaptic connections, was the observation that synaptic density in the molecular layer of the cerebellar cortex was associated with tremor severity [15]. A lower synaptic climbing fiber-Purkinje cell density would subsequently result in a lower degree of inhibition of the dentate nucleus. Furthermore, it has been shown that the excitatory amino acid transporter 2 (EAAT2) is reduced in the cerebellar cortex and increased in the dentate nucleus in patients with ET, indicating that the glutamatergic synaptic transmission is altered [44]. The combined post-mortem observations of these studies showed that the excitatory input (i.e., Glu-input) was disturbed both in the Purkinje cells and at the dentate nucleus in ET patients. Our interpretation is that the observed lower glutamate concentrations (expressed in terms of reduced Glx concentrations) further advocate the role of altered glutaminergic transmission in ET.

### What about the thalamus?

Neurogenic excitatory output from the cerebellum is propagated to the thalamus, which is suggested to have a role in the pathogenesis of ET. Interestingly, the effects of a focal stroke can ameliorate the symptoms, as has been reported by Duncan [45]. Metabolic abnormalities with low NAA/Cr ratio of the contralateral thalamus in ET patients with predominantly tremor of the right arm have been reported [40]. In a recent study, the Glu-concentration was increased in the thalamus of ET patients and correlated to tremor severity, suggesting that thalamic glutamatergic transmission is involved in the pathophysiology [35]. No significant differences in thalamic GABA+, Glx or GABA+/Glx between the ET and HC groups were observed in our study. Furthermore no correlation with GABA+/Glx and tremor severity was seen in the thalamus. The voxel placed in our series is larger and extends beyond the thalamic anatomical borders, thus involving the brain areas in the vicinity of the thalamus (see Fig. 2). Our thalamic results are therefore not easily comparable with previous reports and this may be the reason why our results differ.

### Limitations

Certain experimental aspects of this work were restricted by safety, hardware, partial volume and/or techniques. The patient cohort examined here were all diagnosed by a movement disorder specialist, and then referred to the neurosurgical department, thus they constituted a limited, but very narrow and well-defined group (bilateral, but right-handed tremor). Most of them had severely debilitating hand tremor and they were therefore all selected as being eligible for surgery. Due to the severely disabling disease, surgery was performed immediately after these measurements, with an excellent therapeutic outcome; all patients were also significantly better after surgery (data not shown), indicating the uniquely well-defined cohort.

Unfortunately, the measurements could not be repeated after the surgery, both for ethical and for safety reasons, as the acceptable exposure to electromagnetic fields of the DBS-implants was not compatible with the pre-surgical examination conditions (i.e.*,* 3 T and the SAR level required), and this was a limitation of the present work. At a later time (likely within a few years?) when a sufficiently acceptable set of MR safety conditions have been developed, this may nevertheless be possible to perform.

When performing GABA detection, there are several technical aspects to be considered, one being the complex nature of the spectral scalar coupling pattern of the GABA resonances, and also due to the low signal intensity compared to the other metabolite signals in the spectra. Additionally, another major issue is spectral overlapping by the more intense creatine signal at 3.00 ppm, which obscures the GABA signal at 3.01 ppm severely. In addition, spectral editing is needed to reveal the GABA+ signal, and single voxel MEGA-editing is currently the standard technique. Moreover, as we are quantifying difference spectra (using water scaling in LCModel), a high signal-to-noise ratio (SNR) is necessary; thus, we need a sufficiently large number of signal averages and also a large spectral voxel to achieve a high enough SNR for reliable concentration quantification of the resulting spectra. In comparison with conventional MRS, MEGA-PRESS requires a substantially larger voxel volume, which results in tissue heterogeneity between Purkinje cells and the dentate nucleus etc.

Compared to Louis et al. [23] we used a larger voxel in our cerebellar measurements (35 × 25 × 25 mm^3^, which is equivalent to 21.9 mL compared to 25 × 25 × 25 mm^3^, 15.6 mL). Thus, in our measurements of cerebellar tissue we analyzed part of the cerebellar cortex, in combination with the deep cerebellar nuclei and white matter tracts in the cerebellum. Furthermore, to compensate for the large SNR reduction when using a smaller voxel size, Louis et al. collected more signal averages (196 ON/OFF compared to our 160 ON/OFF). In addition, there might also be other systematic differences between our results since we used different MR systems (Philips compared to Siemens), and different LCModel basis sets were used in the quantification. Our experience is that there are small, but noticeable, systematic differences in spectral analysis when different basis sets are used. Furthermore, Louis et al. used segmentation to determine the tissue composition of their voxel, a procedure which we did not apply. However, our interpretation is that most of these effects are removed when concentration ratios are used, and thus we found a significant correlation between cerebellar GABA+/Glx and ETRS. Although these differences in acquisition and analytical approach exist, the results should in our view still be comparable.

Our present data does not let us to come to a conclusion on whether ET is characterized by such morphological abnormalities in specific cerebellar regions, or whether the biochemical alterations are limited to certain parts, or widespread, as the procedures used here were limited with respect to the spatial resolution of the MRS-technique utilized. However, with increasing advances in MRS technology the voxel volume can be reduced, allowing different regions to be separately analyzed. One such procedure is 3D spiral-MEGA-PRESS GABA Chemical Shift Imaging devised by Bogner et al. [46], for whole brain detection of GABA+ at a spatial resolution of about 1 cm (compared to 3 cm which was used here); the technique requires a uniquely stable spiral acquisition. Another procedure is the Glu-CEST procedure championed by Reddy et al. for detecting Glu-weighted images of the excitatory neurotransmitter with the spatial resolution of conventional imaging [47]. However, both techniques are extremely demanding on subjects, hardware (Glu-CEST requires 7 T) as well as operator skills, and at this time they are only available to selected laboratories.

Since we base our argument on the ratio between GABA+ and Glx, it is of interest to consider what we have measured. The GABA+ measurements include a proportion of macromolecules, and probably also homocarnosine, which is often considered to be a constant fraction (about 40–50%); thus, any short-term changes in GABA+ mainly reflect changes in GABA. Glx is also a composite but of Glu and Gln, although a majority of the signal typically represents Glu [48]. The largest Glu to Gln ratios at the cellular level have been observed in excitatory terminals of parallel fibers in the molecular layer, and also in mossy and climbing fibers, corresponding to a concentration ratio of about 80% Glu (of Glx); and the lowest ratios have been observed in glial cells [49]. Another parameter to consider is that most Gln in the brain is less metabolically active than Glu. Please also note that MRS is typically acquired at a much lower spatial resolution, and the partial volume effects of a wide range of different cell populations then make it harder to predict a neurotransmitter ratio accurately based on cellular concentrations. However, our interpretation is that the majority of the Glx-resonances in the cerebellum are indeed Glu, and this agrees with a report by Goryawala which determined a concentration ratio of between 2 and 4 in gray matter, corresponding to c. 70% Glu (of Glx) in the cerebellum and close 80% in the brain [50].

Determination of absolute concentrations is typically recommended as this will facilitate comparison between different tissues and subjects [22]. Usually, this is achieved by the use of LCModel analysis in combination with water scaling, which is much preferred to creatine-ratios. In the cerebellum, in consideration of the relative tissue heterogeneity, water scaling appears to be less effective due to the spatial (including intra vs. extra-cellular) tissue variation of water content. This is the likely cause of the relatively large variation of the GABA+ and Glx concentrations in Fig. 3a and b. Instead, the uniquely functionally coupled ratio between the related neurotransmitters GABA and Glu will remove the effects of such spatial variation (see Fig. 3c). It is also worth noting that another difference between our study and some others, was the limited number of included ET patients and healthy control subjects (here 10 ET patients and six HCs). However, our patients belonged to a highly selective cohort of ET patients intended for DBS therapy, which may show stronger disease-related parameters.

## Conclusions

This is to our knowledge the first study to show a correlation between the GABA+/Glx ratio in the cerebellum and increasing tremor disability. A change of balance between excitatory and inhibitory neurotransmitters related to a tremor rating scale (ETRS) could explain part of the pathophysiology of ET. This is rather attributed to a decrease in tissue Glu-concentration, than an altered GABA+-concentration, suggesting that an increasing ETRS score is partly due to a disturbance in cerebellar Glu concentration. Importantly, we interpret this as being a consequence of an overall decreased stimulatory effect on the inhibitory Purkinje cells, subsequently leading to decreased influence on nucleus dentatus driving the tremor. This finding may open up a range of entirely new therapeutic alternatives, which so far have been largely unexplored. The exact neurochemical abnormalities underlying ET remain to be elucidated in a larger study.

## Supplementary information

**Additional file 1.**

**Additional file 2.**

**Additional file 3.**

## Data Availability

The MRI and clinical data that support the findings in this study are available on reasonable request from ST, AT and the last author PZ. Raw data are not publicly available due to regulations of the County of Östergötland.
