# Supplementary figures and images for: A pilot study of essential tremor: cerebellar GABA+/Glx ratio is correlated with tremor severity
Source: Cerebellum Ataxias. 2020 Jun 26;7:8. doi: 10.1186/s40673-020-00116-y (PMC7318770; doi:10.1186/s40673-020-00116-y)

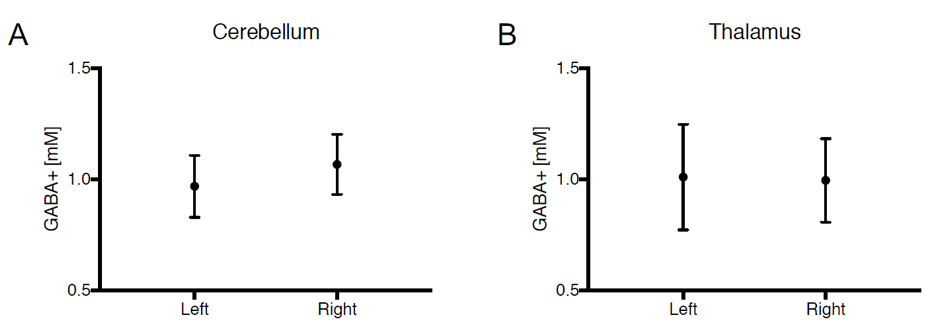

Supplement: Supplementary file 1 — Additional file 1. [file 40673_2020_116_MOESM1_ESM.tif]

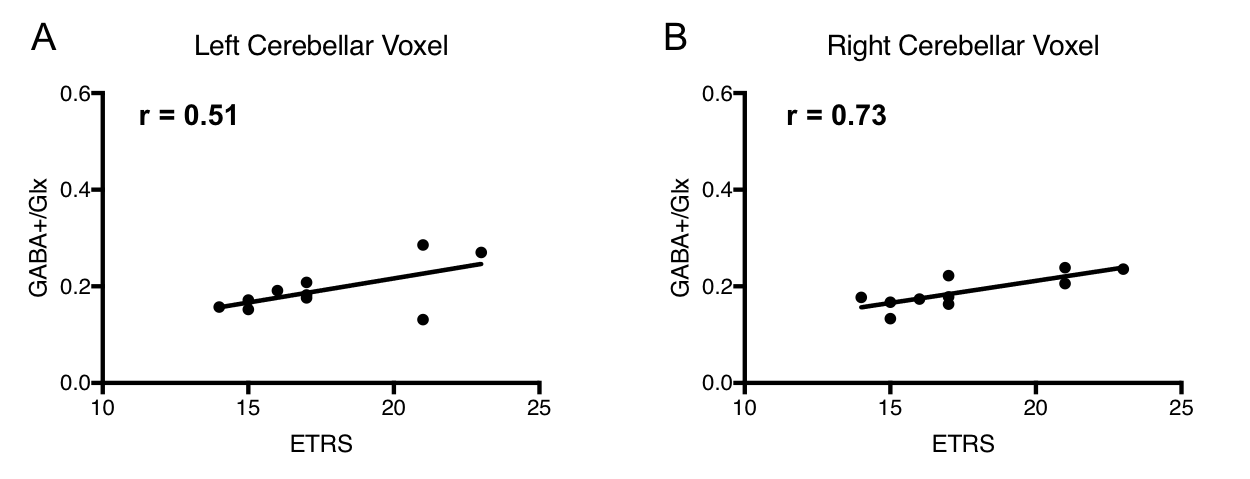

Supplement: Supplementary file 2 — Additional file 2. [file 40673_2020_116_MOESM2_ESM.tif]
