# Supplementary material for: A pilot study of essential tremor: cerebellar GABA+/Glx ratio is correlated with tremor severity
Source: Cerebellum Ataxias. 2020 Jun 26;7:8. doi: 10.1186/s40673-020-00116-y (PMC7318770; doi:10.1186/s40673-020-00116-y)
Supplement: Supplementary file 3 — Additional file 3. [file 40673_2020_116_MOESM3_ESM.docx]

Supplementary table 1. The ETRS score measured for each patient’s right side. The scores from the evaluated items were added to the resulting sum score (maximum score of 32).

| ET Patient | | | 1 | 2 | 3 | 4 | 5 | 6 | 7 | 8 | 9 | 10 |
| --- | --- | --- | --- | --- | --- | --- | --- | --- | --- | --- | --- | --- |
| Item A5 | Right arm | Rest | 0 | 0 | 2 | 0 | 0 | 0 | 1 | 0 | 1 | 0 |
|  |  | Postural | 3 | 2 | 3 | 3 | 2 | 3 | 3 | 3 | 2 | 3 |
|  |  | Intention | 3 | 2 | 3 | 3 | 2 | 3 | 3 | 3 | 2 | 3 |
| Item B10 | Handwriting | | 3 | 2 | 3 | 2 | 2 | 2 | 2 | 3 | 2 | 3 |
| Item B11 | Drawing A | Right | 3 | 2 | 3 | 2 | 2 | 3 | 2 | 2 | 2 | 2 |
| Item B12 | Drawing B | Right | 3 | 2 | 3 | 2 | 2 | 3 | 2 | 2 | 2 | 2 |
| Item B13 | Drawing C | Right | 3 | 2 | 3 | 2 | 2 | 3 | 2 | 2 | 2 | 2 |
| Item B14 | Pouring | Right | 3 | 2 | 3 | 2 | 3 | 4 | 2 | 2 | 2 | 2 |
| Total tremor score (Max 32) | | | 21 | 14 | 23 | 16 | 15 | 21 | 17 | 17 | 15 | 17 |

**
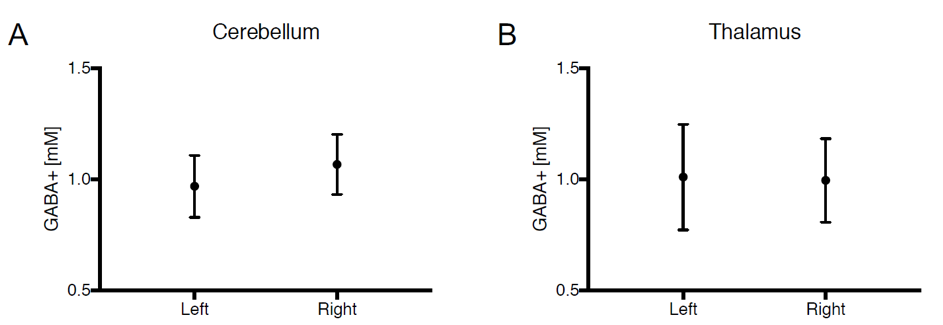
**

**Supplementary figure 1:** **Lateral dependecne of GABA+ concentration.** Mean concentration of GABA+ ± standard deviation for the left and right voxel placements in the cerebellum (A) and the thalamus (B) in essential tremor patients (n = 10).


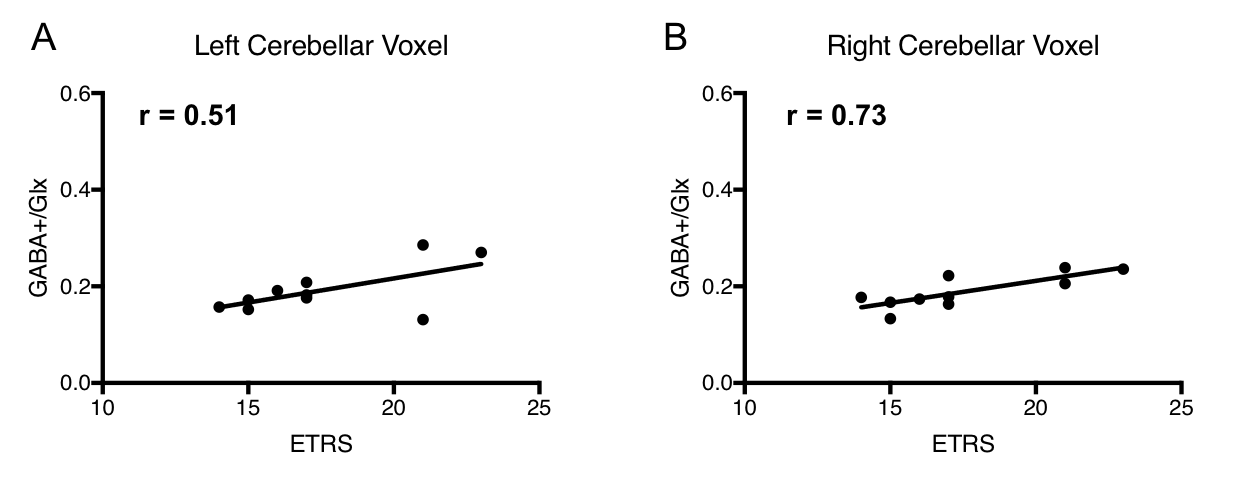


**Supplementary figure 2:** **Concentrations related to ETRS lateral dependence.** Correlation between the cerebellar GABA+/Glx ratio and the ETRS score for the voxel placements in the left (A) and right (B) hemispheres. The Spearman correlation coefficient (r) is reported in each plot.
